# Supplementary material for: Signaling Roleplay between Ion Channels during Mammalian Sperm Capacitation
Source: Biomedicines. 2023 Sep 12;11(9):2519. doi: 10.3390/biomedicines11092519 (PMC10525812; doi:10.3390/biomedicines11092519)
Supplement: Supplementary file 1 [file biomedicines-11-02519-s001.zip › biomedicines-2559542-supplementary.pdf]

Review Methodology

Search Strategy

A systematic review of the literature was carried out on June 2, 2023 by submitting selected keywords into three different databases: PubMed, Scopus, and Web of Science. Search terms used included "Ion channels", "Bicarbonate channels" or "HCO_3^- channels", "Sodium channels" or "Na^+ channels", "Calcium channels" or "Ca^{2+} channels", "Proton channels" or "H^+ channels", "Potassium channels" or "K^+ channels", "Chloride channels" or "Cl^- channels", "Aquaporins", "Spermatozoa" or "Sperm", "Capacitation". A time filter was applied to the search to isolate solely the works published in the last 20 years (2003–2022).

Search Eligibility Criteria

The papers collected from the above searches were screened for the presence of duplicates and narrowed down further using the predefined inclusion and exclusion criteria based on the journal title and abstract. Selected exclusion criteria were (1) the paper is not written in English, (2) the paper is not an original research paper (review articles, book chapters, editorials, conference abstracts, and letters; studies with no abstracts were eliminated), or (3) the paper is not freely available (using the institutional credentials of the Slovak University of Agriculture). The resulting list was then subjected to an inclusion round in which we considered only original laboratory research studies, conducted on spermatozoa as a model, that met our original search aims. All papers that did not discuss the involvement of ion channels in sperm activation in the title or abstract were excluded. In summary, the inclusion criteria were (1) the paper covers original laboratory research; (2) spermatozoa are the model of the study; and (3) the paper is relevant based on its title and abstract.

Article Selection and Processing

The final list obtained by this iterative selection process was independently reviewed by three different co-authors of the paper. The full text was obtained for each of the included articles. If an article was not readily available, the corresponding author was personally contacted, and the manuscript of the relevant study was obtained. No papers were excluded in this step.

Search Results

The initial search yielded 800 titles from PubMed, 426 titles from Scopus, and 611 titles from Web of Science, for a total of 1 837. The search results were collated, and subsequent removal of duplicates reduced this number to 1 269. The exclusion round led to the rejection of 436 reviews, editorials, book chapters, articles not written in English or unavailable papers. The final round of rejection eliminated 284 articles that did not meet the inclusion criteria. The final number of articles that had met the pre-established eligibility criteria was and the subsequent exclusion and inclusion rounds led to the final number of 549 articles with information that was considered for the purposes of this review.
